# Development of Predictive Models Based on Biochemical Parameters to Triage COVID-19 Patients: A Study Conducted in a Tertiary Care Hospital

**DOI:** 10.7759/cureus.56197

**Published:** 2024-03-14

**Authors:** Mainak Sinha, Ayan Banerjee, Sushil Kumar, Mala Mahto, Bandana Kumari, Alok Ranjan, Akash Bansal

**Affiliations:** 1 General Surgery, All India Institute of Medical Sciences, Patna, IND; 2 Biochemistry, All India Institute of Medical Sciences, Patna, IND; 3 Community and Family Medicine, All India Institute of Medical Sciences, Patna, IND; 4 Biochemistry, All India Institute of Medical Sciences, Gorakhpur, IND

**Keywords:** interleukin (il)-6, machine learning, python, predictive models, procalcitonin, covid 19

## Abstract

Background

The COVID-19 disease continues to cause severe mortality and morbidity. Biochemical parameters are being used to predict the severity of the infection. This study aims to predict disease severity and mortality to help reduce mortality through timely intervention in a cost-effective way.

Methods

A total of 324 COVID-19 cases admitted at our hospital (All India Institute of Medical Sciences, Patna, BR, India) between June 2020 to December 2020 (phase 1: 190 patients) and April 2021 to May 2021 (phase 2: 134 patients) were recruited for this study. Statistical analysis was done using SPSS Statistics version 23 (IBM Corp., Armonk, NY, USA) and model prediction using Python (The Python Software Foundation, Wilmington, DE, USA).

Results

There were significant differences in biochemical parameters at the time of admission among COVID-19 patients between phases 1 and 2, ICU and non-ICU admissions, and expired and discharged patients. The receiver operating characteristic (ROC) curves predicted mortality solely based on biochemical parameters. Using multiple logistic regression in Python, a total of four models (two each) were developed to predict ICU admission and mortality. A total of 92 out of 96 patients were placed into the correct management category by our model. This model would have allowed us to preserve 17 of the 21 patients we lost.

Conclusions

We developed predictive models for admission (ICU or non-ICU) and mortality based on biochemical parameters at the time of admission. A predictive model with a significant predictive capability for IL-6 and procalcitonin values using normal biochemical parameters was proposed. Both can be used as machine learning tools to prognosticate the severity of COVID-19 infections. This study is probably the first of its kind to propose triage for admission in the ICU or non-ICU at the medical emergency department during the first presentation for the necessary optimal treatment of COVID-19 based on a predictive model.

## Introduction

The devastating effects of COVID-19, which emerged from the newly discovered SARS-CoV-2, have been felt all over the world. The virus emerged in Wuhan, China, in December 2019 and spread worldwide, causing severe morbidity and mortality [1,2]. The disease causes mild to severe lower respiratory tract symptoms [3]. This condition was initially poorly understood, making diagnosis and treatment difficult. Based on clinical signs and saturation of peripheral oxygen (SpO2), the WHO classified COVID-19 as mild, moderate, or severe [4]. The WHO's severity classification is simple but does not convincingly predict mortality among COVID-19 patients. Biochemical parameters such as IL-6, ferritin, D-dimer, and procalcitonin are used to assess the severity of the COVID-19 infection. Our study aims to develop a model based on these laboratory biochemical parameters to predict admission criteria (ICU or non-ICU) as well as mortality at the initial presentation of COVID-19-infected patients in the ED. Both IL-6 and procalcitonin are established biochemical markers of COVID-19 severity [5-11]. However, the primary health centers (PHCs) of developing countries lack equipment for measuring IL-6 and procalcitonin. Therefore, we tried to create a model to predict the values of IL-6 and procalcitonin from routine biochemical investigations such as serum urea, neutrophil lymphocyte ratio, albumin, alanine transferase (ALT), and lactate dehydrogenase (LDH), among others. This research will help in the triage and management of future COVID-19 cases. These models can be used in machine learning to predict the severity and mortality of future patients in pandemics related to COVID-19.

## Materials and methods

Study design and setting

A retrospective observational study was conducted in a tertiary care hospital (All India Institute of Medical Sciences, Patna, BR, India), where levels of various analytes, i.e., serum electrolytes of sodium (Na) and calcium (Ca), parameters of liver function tests (LFT), kidney function tests (KFT), LDH, IL-6, procalcitonin, ferritin, C-reactive protein (CRP), and neutrophil lymphocyte (NL) ratio, were collected from COVID-19-positive patients who were admitted during the first (June 2020 to December 2020; referred to in this study as phase 1) and second wave of the pandemic (April 2021 to May 2021; referred to as phase 2). The LFT, KFT, CRP, and LDH were measured in the Beckman Coulter AU680 (Beckman Coulter Inc., Pasadena, CA, USA), and IL-6 and ferritin in the Siemens Advia Centaur (Siemens Healthineers, Erlangen, BY, DEU) per the manufacturer's protocol. Procalcitonin was measured in the Ortho Clinical Diagnostics Vitros 5600 (QuidelOrtho, San Diego, CA, USA). This study examined 14 biochemical parameters, namely NL ratio, D-dimer, international normalized ratio (INR), Na, Ca, creatinine, urea, albumin, LDH, CRP, ALT, ferritin, procalcitonin, and IL-6. All patient-related data was collected from the hospital's information system.

Inclusion and exclusion criteria

Patients who tested positive for COVID-19 via reverse transcription-polymerase chain reaction (RT-PCR) and were admitted to our hospital (both ICU and non-ICU) during our study period (phase 1 and phase 2) were included in our study. During phases 1 and 2, COVID-19-positive patients were admitted per their severity classification. Mild included those patients with symptoms such as fever, cough, and sore throat with a respiratory rate ≤ 24 per minute and SpO2 > 94% (range: 94% to 100%) on room air or 200 < partial pressure of oxygen (PaO2) in arterial blood/fraction of inspired oxygen (FiO2) < 300 mmHg. Those in the moderate category have clinical features of dyspnea and/or hypoxia with a respiratory rate > 24 per minute and SpO2 ≥ 90% on room air, or PaO2/FiO2 = 100 to 200 mmHg. The severe category is characterized by clinical signs of pneumonia and any one of the following: respiratory rate > 30 breaths/min, severe respiratory distress, SpO2 < 90% on room air, or PaO2/FiO2 ≤ 100 mmHg. The severe category of patients were admitted to the ICU, and the mild and moderate category of patients were admitted to the non-ICU (ward/high dependency unit (HDU)) for treatment [4]. The COVID-19-negative patients admitted during the study period due to any other cause were not included in this study.

Statistical analysis

For data analysis, SPSS Statistics version 23 (IBM Corp., Armonk, NY, USA) was used. The prediction model was built using Python version 3.6 (The Python Software Foundation, Wilmington, DE, USA). We used logistic regression for ICU admission and mortality prediction. Categorical variables were presented as proportions. Continuous variables were presented as median and interquartile range values. All parameters were tested for normality using the Shapiro-Wilk test. Since all biochemical variables were non-normally distributed, Mann-Whitney U-tests were used for continuous variables and chi-square tests for categorical variables. Spearman's correlation was used to assess the relationship between ICU admission and mortality using biochemical variables taken at admission. Receiver operating characteristic (ROC) curve analysis determined the optimum cut-off points of significantly correlated biochemical variables with disease severity and area under the curve, with a 95% confidence interval and significance value for each variable. The index of union determined the cut-off point. A two-tailed p-value < 0.05 was considered significant.

For designing the prediction model using Python, we predicted ICU admission and mortality status using input variables. The dichotomous variable was predicted using logistic regression. Two input variables, the feature importance coefficient, and the correlation coefficient, were used in model prediction. Using feature importance selection, we selected only input variables that predicted ICU admission and mortality. We chose input variables highly correlated with the ICU using the correlation coefficient. Table 1 shows the correlation coefficients and feature importance coefficients of biochemical parameters.

**Table 1:** Correlation and feature importance coefficient of biochemical parameters NL ratio: Neutrophil to lymphocyte ratio, INR: International normalized ratio, Na: Sodium, Ca: Calcium, ALT: Alanine transaminase, LDH: Lactate dehydrogenase, CRP: C-reactive protein

Dependent variable	Biochemical parameters	Spearman’s correlation coefficients	p-value	Feature importance coefficients
ICU admission	NL ratio	0.378	0.001	0.24722
	D-dimer	0.283	0.001	-0.0235
	INR	0.116	0.059	0.50525
	Na	0.058	0.302	-0.1471
	Ca	-0.112	0.044	0.12298
	Creatinine	0.094	0.094	-0.1773
	Urea	0.287	0.001	-0.0458
	ALT	0.061	0.274	0.30363
	Albumin	-0.293	0.001	0.56469
	LDH	0.379	0.001	0.09141
	IL-6	0.391	0.001	0.15779
	CRP	0.236	0.001	-0.3039
	Ferritin	0.167	0.01	-0.0747
	Procalcitonin	0.323	0.001	-0.1595
Mortality	NL ratio	0.368	0.001	0.07725
	D-dimer	0.303	0.001	-0.396
	INR	0.105	0.087	-0.2753
	Na	0.095	0.088	-0.3938
	Ca	-0.129	0.021	0.31741
	Creatinine	0.125	0.025	-0.288
	Urea	0.363	0.001	0.06753
	ALT	0.092	0.099	0.2652
	Albumin	-0.347	0.001	-0.0137
	LDH	0.504	0.001	0.03879
	IL-6	0.389	0.001	0.29913
	CRP	0.209	0.001	-0.4541
	Ferritin	0.2	0.002	-0.307
	Procalcitonin	0.337	0.001	-0.0331

We calculated all performance metrics to evaluate our model. Our input variable model covers both groups (ICU and non-ICU). Our model produced Z log (odds of in ICU/mortality). The sigmoid function calculates probability from log odds Z. The sigmoid function describes this transformation: ICU probability = 1 / (1 + e^(-Z)). In our dataset, if the probability of ICU admission was greater than 50%, the patient should be admitted to the ICU for better outcomes, and if the mortality probability was greater than 50%, the patient had a very high mortality risk.

We constructed ROC curves in SPSS Statistics based on the probabilities we got from the ICU and death prediction models (based on the correlation coefficient). From that ROC curve, we got the cutoff values for ICU admission and mortality. For ICU admission, the probability cutoff value was 40.64%, and for mortality, it was 51.14%, but in our dataset, we have taken the cutoff probabilities as 50% for both models. As we chose 50% probability, the Brier score is (0.50−1)2 = (0.50−0)2 = 0.25. By increasing the ICU admission cutoff value, we increased the specificity of our model so that there would be less waste of ICU beds, considering the scarcity of ICU beds. By decreasing the mortality cutoff value, we increased the sensitivity of our model to prevent mortality as much as possible.

We also predicted IL-6 and procalcitonin using low-resource input variables. Multiple linear regression predicts the outcome because all output variables are continuous. The correlation coefficient lets us find the input variables for each output variable. We chose only highly correlated input variables for each output variable using the correlation coefficient. We then calculated all performance metrics to see how our model performed for each case.

## Results

Out of 324 patients, 190 in phase 1 and 134 in phase 2 were included in our study. Table 2 shows 26.3% phase 1 mortality and 52.9% phase 2 mortality, which was statistically significant. Phase 1 had 27.89% ICU admissions, and phase 2 had 54.48%. Table 2 compares the biochemical parameters of phase 1 and phase 2 COVID-19 patients at admission; the NL ratio, Na, Ca, CRP, and procalcitonin differed significantly between phases 1 and 2. Sodium, Ca, and procalcitonin median values were normal. Phase 2 had higher NL ratios and lower CRPs than in phase 1.

**Table 2:** Demographic details and comparison between the biochemical parameters of phases 1 and 2 The 'n' denotes the number of samples taken into consideration for analysis of that particular parameter. All values except age are median and range. NL ratio: Neutrophil to lymphocyte ratio, Na: Sodium, Ca: Calcium, CRP: C-reactive protein, ALT: Alanine transaminase, INR: International normalized ratio

Variables		Phase 1 (n = 190)	Phase 2 (n = 134)	p-value
Gender	Male	150 (79%)	96 (71.6%)	0.13
	Female	40 (21%)	38 (28.4)	
Age (in years)		52.98+/-17.85	54.29+/-15.09	0.477
Age group	<15	4 (2%)	0 (0%)	0.153
	15 to 30	19 (10%)	6 (4.5%)	
	31 to 15	34 (18%)	32 (23.9%)	
	46 to 60	52 (27.4%)	42 (31.3%)	
	60 to 75	62 (32.6%)	44 (32.8%)	
	≥ 75	19 (10%)	10 (7.5%)	
Status	Discharge	140	63	0.0001
	Death	50 (26.3%)	71 (52.9%)	
ICU admission	Yes	53 (28 %)	73 (55%)	0.0001
	No	137 (72%)	61 (45%)	
Duration of hospital stay (in days)		15 (2 to 33)	12 (6 to 26)	0.0868
Differences in biochemical parameters taken at the time of admission (normal range)	NL Ratio (1-3)	6.55 (3.61-13) (n = 189)	9.89 (4.54-18.38) (n = 131)	0.0139
	Na (135-145 meq/L)	134.8 (131-137) (n = 189)	137.13 (133.7-140.31) (n = 133)	0.001
	Ca (8.6-10 meq/L)	8.5 (8.1-8.9) (n = 189)	8.63 (8.31-9.05) (n = 133)	0.0335
	CRP (0-5 mg/L)	98 (48-159.07) (n = 129)	59.51 (22.99-125.03) (n = 113)	0.0291
	Procalcitonin (<0.5 ng/ml)	0.205 (0.0-0.675) (n = 100)	0.138 (0.074-0.319) (n = 123)	0.03
Differences in biochemical parameters taken at the time of discharge (normal range)	NL ratio(1-3)	4.222 (3.394-5.169) (n = 125)	6.831 (4.673-8.451) (n = 56)	0.012
	Na (135-145 meq/L)	136 (135-137) (n = 129)	137.36 (136.1-138.31) (n = 56)	0.041
	ALT (13-40 U/L)	51.4 (40.3-66) (n = 115)	91.7(59.7-123) (n = 55)	0.001
Differences in biochemical parameters taken just before death (normal range)	INR (1.00)	1.24 (1.1-1.5) (n = 25)	1.1(1.04-1.17) (n = 44)	0.007
	Creatinine (0.7-1.3 mg/dl)	2.5 (1.7-3.6) (n = 43)	1.02 (0.8-1.55) (n = 68)	0.001
	Urea (13-43 mg/dl)	135.4 (100.8-177.7) (n = 43)	69.55 (58-112.6) (n = 68)	0.006
Differences in biochemical parameters taken at the time of admission among the deceased patients (normal range)	Na (135-145 meq/L)	133.3 (130-138.6) (n = 50)	138 (134.7-142) (n = 70)	0.0001
	Ferritin (22-322 ng/ml)	629 (303.85-1067) (n = 40)	766.48 (591.25-1650) (n = 55)	0.0454
	Procalcitonin (<0.5 ng/ml)	0.04 (0.2-1.53) (n = 32)	0.194 (0.093-0.739) (n = 65)	0.0116

Biochemical parameters between phases 1 and 2 taken at the time of discharge were compared. Three parameters showed significant differences: NL ratio, Na, and ALT. Both phases had normal Na levels, but phase 2 discharges had a higher NL ratio and ALT than phase 1 discharges.

We compared biochemical parameters at the time of admission in phases 1 and 2 among the deceased patients. In both phases, Na, ferritin, and procalcitonin showed significant differences. Phase 2 had higher median values for all three parameters.

A comparison of phase 1 and phase 2 biochemical parameters in the last drawn blood samples among deceased patients was made. The INR, creatinine, and urea were the only three parameters out of 14 that showed statistically significant differences. Both phases exhibited a normal INR. In phase 1 fatalities, the levels of creatinine and urea were higher than in phase 2 deaths.

Table 3 compares biochemical parameters for ICU and non-ICU patients taken at the time of admission. Ten of the 14 parameters differed significantly between ICU and non-ICU admissions. The ICU patients had a higher NL ratio, D-dimer, urea, LDH, IL-6, CRP, procalcitonin, and ferritin than non-ICU patients. Also, the ICU patients had lower albumin and Ca.

**Table 3:** Comparison of biochemical parameters between ICU and non-ICU patients at the time of admission The 'n' denotes the number of samples taken into consideration for analysis of that particular parameter. All values are median and range. NL ratio: Neutrophil to lymphocyte ratio, Ca: Calcium, CRP: C-reactive protein, INR: International normalized ratio

Biochemical parameters at admission (normal range)	Non-ICU patients	ICU patients	p-value
NL ratio (1-3)	5.183 (4.549-6.308) (n = 194)	12.133 (10.295-14.219) (n = 126)	0.001
D-dimer (<0.2 microgram/ml)	0.8(0.69-0.97) (n = 117)	1.35 (1.11-1.6) ( n = 100)	0.001
Ca (8.6-10 meq/L)	8.63 (8.55-8.73) (n = 197)	8.5(8.4-8.6) (n = 125)	0.045
Urea (13-43 mg/dl)	32 (30-35.1) (n = 197)	46.9 (42.4-52) (n = 125)	0.001
Albumin (3.4-4.8 g/dl)	3.6 (3.5-3.7) (n = 197)	3.305 (3.28-3.4) (n = 124)	0.001
LDH (230-460 U/L)	808.52 (728-877) (n = 128)	1169.7 (1101.3-1239.84) (n = 103)	0.001
IL-6 (<6.4 pg/ml)	9.8 (6.2-13.3) (n = 59)	33.8 (23.9-54.2) (n = 87)	0.001
CRP (0-5 mg/L)	62.4 (48.76-89.2) (n = 140)	113.86 (83.9-125.03) (n = 101)	0.001
Ferritin (22-322 ng/ml)	584.4 (403.3-696.1) (n = 141)	744.83 (605.2-783.69) (n = 99)	0.01
Procalcitonin (<0.5 ng/ml)	0.103 (0.1-0.142) (n = 123)	0.230 (0.19-0.4) (n = 100)	0.001
Biochemical parameters at discharge (normal range)	Non-ICU patients	ICU patients	p-value
INR (1.00)	0.915 (0.9-1) (n = 56)	1.1 (0.9-1.29) (n = 8)	0.029
Ca (8.6-10 meq/L)	8.8 (8.65-8.9) (n = 156)	8.5 (8.2-8.9) (n = 26)	0.032
Ferritin (22-322 ng/ml)	328.5 (225-573) (n = 55)	524.55 (378.2-865.9) (n = 12)	0.038
Procalcitonin (<0.5 ng/ml)	0.1 (0.03-0.2) (n = 33)	0.37 (0.1-2.9) (n = 8)	0.017

Four biochemical parameters showed a significant difference between ICU and non-ICU patients at the time of discharge, while INR, Ca, and procalcitonin were normal. The ICU patients had higher ferritin levels than non-ICU patients.

Table 4 shows the comparison between the biochemical parameters taken at the time of admission in COVID-19 patients who succumbed to the disease and those who were discharged. Eleven of the 14 parameters showed a significant difference between those discharged and those who expired. The NL-ratio, D-dimer, urea, LDH, IL-6, CRP, and ferritin were significantly higher in deceased patients. Though within normal limits, discharged and deceased patients had significant differences in Ca, creatinine, albumin, and procalcitonin. Deceased patients had higher creatinine and procalcitonin and lower Ca and albumin when compared with the discharged patients.

**Table 4:** Comparison of biochemical parameters between patients who survived and patients who succumbed to the disease NL ratio: Neutrophil to lymphocyte ratio, Ca: Calcium, LDH: Lactate dehydrogenase, CRP: C-reactive protein, INR: International normalized ratio, Na: Sodium The 'n' denotes the number of samples taken into consideration for analysis of that particular parameter. All values are median and range.

Biochemical parameters at the time of admission (normal range)	Discharged	Deceased	p-value
NL ratio (1-3)	5.259 (4.647-6.417) (n = 199)	12.521 (10.464-14.833) (n = 121)	0.001
D-dimer (<0.2 microgram/ml)	0.82 (0.69-1) (n = 125)	1.38 (1.11-1.68) (n = 92)	0.001
Ca (8.6-10 meq/L)	8.665 (8.555-8.79) (n = 202)	8.5 (8.4-8.6) (n = 120)	0.021
Creatinine (0.7-1.3 mg/dl)	0.84 (0.8-0.9) (n = 202)	0.9 (0.87-0.97) (n = 120)	0.025
Urea (13-43 mg/dl)	31.45 (30-35) (n = 202)	50.15 (44.8-57.9) (n = 120)	0.001
Albumin (3.4-4.8 g/dl)	3.6 (3.5-3.7) (n = 201)	3.3 (3.24-3.4) (n = 120)	0.001
LDH (230-460 U/L)	779 (706-843) (n = 131)	1216.735 (1146.5-1338.6) (n = 100)	0.001
IL-6 (<6.4 pg/ml)	10.5 (6.6-13.3) (n = 66)	36.3 (25.2-54.8) (n = 80)	0.001
CRP (0-5 mg/L)	62 (48.76-89.2) (n = 149)	103.75 (83.9-119.06) (n = 92)	0.001
Ferritin (22-322 ng/ml)	528.4 (375.5-669.6) (n = 145)	745.24 (645-841) (n = 95)	0.002
Procalcitonin (<0.5 ng/ml)	0.104 (0.1-0.142) (n = 126)	0.23 (0.194-0.4) (n = 97)	0.001
Biochemical parameters in non-ICU patients at the time of admission (normal range)	Discharged	Deceased	p-value
NL ratio (1-3)	5.012 (4.358-5.857) (n = 173)	7.909 (4.731-16.316) (n = 21)	0.029
Ca (8.6-10 meq/L)	8.7 (8.6-8.8) (n = 176)	8.4 (8.1-8.7) (n = 21)	0.037
Urea (13-43 mg/dl)	30.45 (28.2-33) (n = 176)	55.9 (38-104.4) (n = 21)	0.001
Albumin (3.4-4.8 g/dl)	3.615 (3.51-3.72) (n = 176)	3.3 (3.01-3.5) (n = 21)	0.001
LDH (230-460)	777.4 (706-835.99) (n = 114)	1288.1 (852.5-1829) (n = 14)	0.001
CRP (0-5 mg/L)	55 (44.87-80) (n = 126)	100.25 (69.07-205.1) (n = 14)	0.031
Ferritin (22-322 ng/ml)	526 (359.8-658.9) (n = 125)	927.25 (431-1650) (n = 16)	0.029
Procalcitonin (<0.5 ng/ml)	0.1 (0.086-0.119) (n = 110)	0.6 (0.2-1.56) (n = 13)	0.001
Biochemical parameters in discharged/deceased non-ICU patients (normal range)	Discharged	Deceased	p-value
NL ratio (1-3)	4.673 (3.854-6) (n = 155)	23.5 (7.636-35.148) (n = 13)	0.001
D-dimer (<0.2 microgram/ml)	0.52 (0.4-0.69) (n = 62)	9.665 (2.03-17.3) (n = 2)	0.034
INR (1.00)	0.915 (0.9-1) (n = 56)	1.15 (1.04-5.3) (n = 6)	0.002
Na(135-145 meq/L)	136.1 (135.64-137) (n = 159)	143.2 (130-153.9) (n = 54)	0.018
Ca (8.6-10 meq/L)	8.8 (8.65-8.9) (n = 156)	8.15 (6.5-9) (n = 14)	0.003
Creatinine (0.7-1.3 mg/dl)	0.765 (0.7-0.8) (n = 158)	2.45 (1.01-5.9) (n = 14)	0.001
Urea(13-43 mg/dl)	32(29.3-34.8) (n=157)	151.5(77.2-186) (n=14)	0.001
Albumin (3.4-4.8 g/dl)	3.4 (3.3-3.5) (n = 150)	2.7 (2.1-3.1) (n = 10)	0.001
LDH (230-460 U/L)	686.41 (579-754) (n = 53)	1896.65 (554-2684.3) (n = 6)	0.002
CRP (0-5 mg/L)	9.785 (5.06-15.32) (n = 76)	166 (65.6-378.4) (n = 7)	0.001
Ferritin (22-322 ng/ml)	328.5 (225-573) (n = 55)	1399.5 (318.8-1650) (n = 6)	0.002
Procalcitonin (<0.5 ng/ml)	0.1 (0.03-0.2) (n = 33)	2.17 (0.2-15.4) (n = 4)	0.007
Biochemical parameters in ICU patients at the time of admission (normal range)	Discharged	Deceased	p-value
Na (135-145 meq/L)	133.7 (131-135) (n = 26)	136.48 (135.16-138.51) (n = 99)	0.032
Urea (13-43 mg/dl)	39.55 (32-48) (n = 26)	49.2 (43.1-57.9) (n = 99)	0.029
LDH (230-460 U/L)	795 (665-1079) (n = 17)	1199.175 (1145.72-1318.5) (n = 89)	0.002
Biochemical parameters in discharged/deceased ICU patients (normal range)	Discharged	Deceased	p-value
NL ratio (1-3)	6.7 (3.363-9.111) (n = 26)	27.971 (21.295-31.667) (n = 97)	0.001
D-dimer (<0.2 microgram/ml)	0.62 (0.3-1.8) (n = 8)	3.05 (2.5-4.92) (n = 60)	0.001
Na (135-145 meq/L)	137 (134.9-138) (n = 26)	142.135 (140.73-144.26) (n = 98)	0.001
Ca (8.6-10 meq/L)	8.5 (8.2-8.9) (n = 26)	7.59 (7.4-7.87) (n = 98)	0.001
Creatinine (0.7-1.3 mg/dl)	0.7 (0.5-0.9) (n = 26)	1.4 (1.01-2.05) (n = 97)	0.002
Urea (13-43 mg/dl)	31.8 (25-38) (n = 26)	92.5 (67.6-113.2) (n = 97)	0.001
Albumin (3.4-4.8 g/dl)	3.13 (2.8-3.7) (n = 22)	2.5 (2.4-2.66) (n = 93)	0.001
LDH (230-460 U/L)	697.5 (434-940) (n = 11)	1271.3 (1121.58-1441.94) (n = 56)	0.001
IL-6 (<6.4 pg/ml)	5.65 (2.3-8.4) (n = 4)	99.05 (47.9-155.2) (n = 64)	0.001
CRP (0-5 mg/L)	2.85 (2.8-26) (n = 12)	119.17 (84.82-142.05) (n = 61)	0.001

The COVID-19 patients admitted to non-ICUs were compared for biochemical parameters taken at the time of admission. Eight of the 14 parameters showed a significant difference between discharged and deceased patients. Deceased patients had a higher NL-ratio, urea, LDH, CRP, and ferritin. Though within the normal range, Ca, albumin, and procalcitonin values differed between the two groups. Deceased patients had higher procalcitonin and lower Ca and albumin.

We compared biochemical parameters taken at the time of discharge or death between discharged and deceased patients admitted to the non-ICU. Twelve parameters showed a significant difference. Deceased patients had a higher NL ratio, D-dimer, creatinine, urea, LDH, CRP, ferritin, and procalcitonin than discharged patients. Calcium and albumin were lower in deceased patients, but INR and Na were higher.

We contrasted the biochemical parameters measured at the time of ICU admission between the deceased and the discharged patients. Sodium, urea, and LDH are three biochemical parameters that differed significantly between patients who were discharged and those who died. The Na levels of both discharged and deceased patients were within the normal range. Patients who died had substantially higher levels of urea and LDH than patients who were discharged.

We also compared biochemical parameters taken at the time of discharge or death between discharged and deceased ICU patients. Ten of the 14 parameters showed a significant difference between those who were discharged and those who died. Deceased patients had higher NL-ratio, D-dimer, creatinine, urea, LDH, IL-6, and CRP values. Deceased patients had lower Ca and albumin levels and higher Na levels.

Many hospitals lack specialized investigations. Thus, using routinely tested parameters, we attempted to predict baseline IL-6 and procalcitonin. We calculated IL-6 and procalcitonin values using simple variables: (A) IL-6 = 559.1004 + (1.2146*NL ratio) - (10.6273*Ca) - (0.1128*urea) - (0.3161*ALT)-(121.3760*albumin) + (0.0247*LDH) (p-value-4.91e-06; R squared: 0.244). And (B) procalcitonin = 1.6856 + (0.0133*NL ratio) - (0.0437*D-dimer) + (0.2965*INR) + (1.4655*creatinine) - (0.0284*Urea) - (0.6847*albumin) + (0.0010*LDH) (p-value-0.00692; R squared: 0.349)

**Figure 1:**
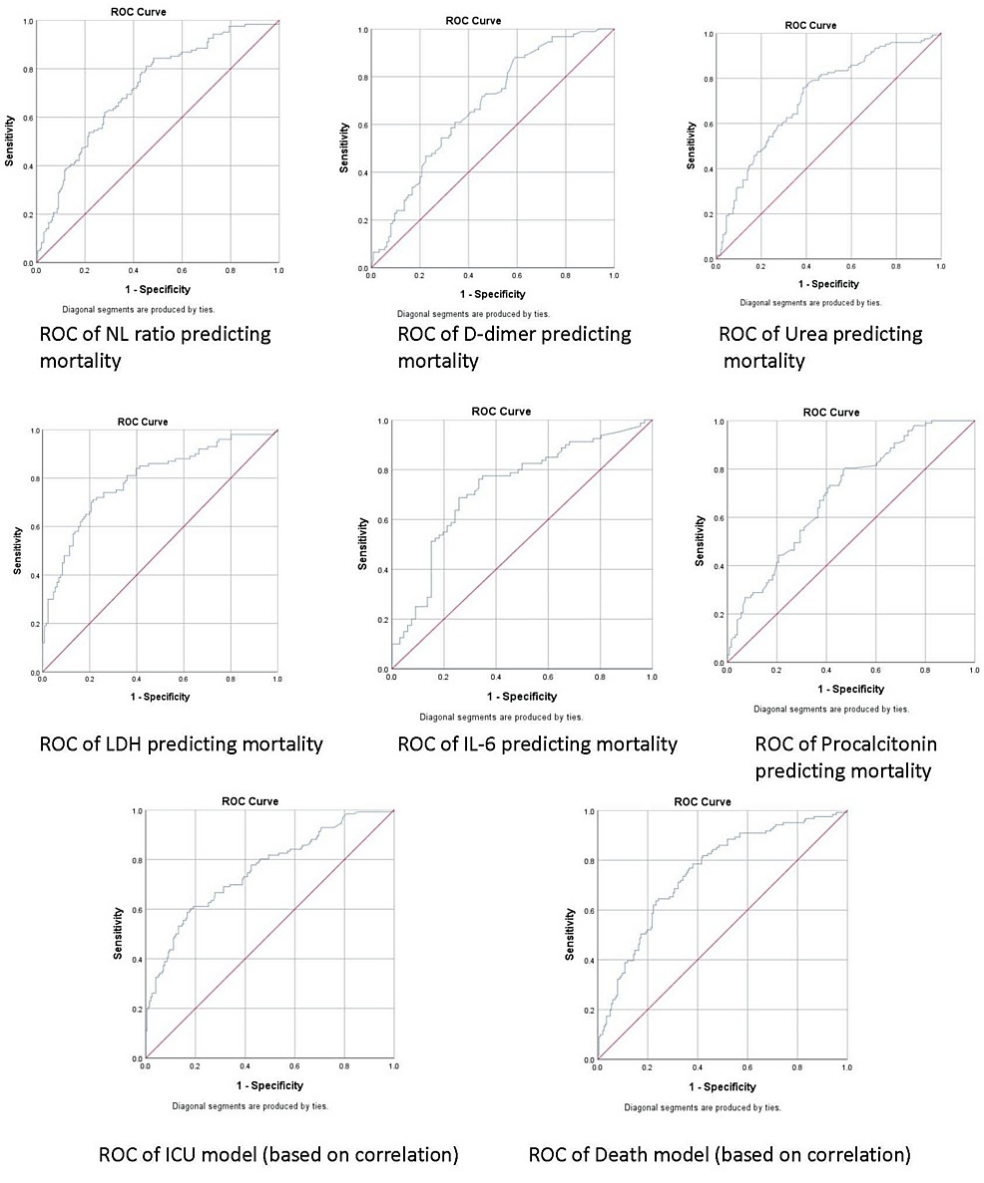
ROC curves of parameters predicting mortality and ICU admission ROC: Receiver operating characteristic

Next, we wanted to determine a specific cutoff value for the biochemical parameters that can be used to predict the severity of the disease category, independent of the clinical categorization. Therefore, the ROC curves of significantly associated biochemical variables were determined (Table 5, Figure 1).

**Table 5:** Cut-off values of biochemical parameters to predict mortality and ICU admission NL ratio: Neutrophil to lymphocyte ratio, LDH: Lactate dehydrogenase

Variable	Cut-off	Sensitivity	Specificity	Accuracy (maximum)	Area under the curve (in %)	p-value
NL ratio	11.56	53.72%	78.39%	69%	71.9	0.0001
D-dimer	1.58	46.74%	77.6%	64.52%	67.7	0.0001
Urea	55.2	45.83%	83.17%	69.25%	72	0.0001
LDH	1089	70%	79.39%	75.32%	79	0.0001
IL-6	16.4	76.25%	66.67%	71.92%	73	0.0001
Procalcitonin	0.33	44.33%	79.37%	64.13%	70	0.0001
ICU model (based on correlation)	40.64	69%	68.7%	77+/-5%	75.6	0.0001
Mortality model (based on correlation)	51.14	68.6%	69.3%	77+/-5%	75.1	0.0001

Clinical parameters are used to categorize patients into ICU or non-ICU admissions. However, we have used biochemical parameters to predict ICU admission and mortality in COVID-19 patients. To do so, we have developed four predictive models based on the values of biochemical parameters taken at the time of admission and created two models each to determine ICU admission and mortality.

The first ICU prediction model (M1 ICU) used the parameter feature importance. The accuracy was 82+/-5%. Our logistic regression equation is Z = B0+B1X1+B2X2+B3X3+B4X4+B5X5. The second model (M2 ICU) uses biochemical parameter correlation coefficients with ICU admission. The accuracy was 77+/-5%. Our logistic regression equation is Z = B0+B1X1+B2X2+B3X3.

Our first mortality prediction model (M1 Mortality) uses parameter feature importance. The accuracy was 74+/-5%. Our logistic regression equation is Z = B0+B1X1+B2X2+B3X3+B4X4+B5X5. The second model (M2 Mortality) uses biochemical parameter correlation coefficients with mortality. The accuracy was 77+/-5%. Our logistic regression equation is Z = B0+B1X1+B2X2+B3X3+B4X4. The details of these models are given in Table 6.

**Table 6:** Predictive models for ICU and non-ICU admission and mortality NL ratio: Neutrophil to lymphocyte ratio, INR: International normalized ratio, ALT: Alanine transaminase, CRP: C-reactive protein, LDH: Lactate dehydrogenase, Na: Sodium, Ca: Calcium, PPV: Positive predictive value, NPV: Negative predictive value

Predictive models	Logistic regression equation	Intercept	Variables	Coefficients	Accuracy
ICU admission based on feature importance	Z = B0+B1X1+B2X2+B3X3+B4X4+B5X5 (M1 ICU)	(B0): (0.292)	X1:NL ratio	B1: 0.06	Accuracy 82+/-5%
			X2:INR	B2: -0.575	
			X3:ALT	B3: 0.012	
			X4:Albumin	B4: -0.439	
			X5:CRP	B5: 0.012	
ICU admission based on correlation coefficients	Z = B0+B1X1+B2X2+B3X3 (M2 ICU)	(B0): (-0.143)	X1:Albumin	B1: -0.682	Accuracy 77+/-5%; sensitivity 69%; specificity 68.7%; PPV 58.4%; NPV 77.7%
			X2:LDH	B2: 0.003	
			X3:IL-6	B3: 0.012	
Mortality based on feature importance	Z = B0+B1X1+B2X2+B3X3+B4X4+B5X5 (M1 Mortality)	(B0): (0.022)	X1:D-dimer	B1: 0.812	Accuracy 74+/-5%
			X2:Na	B2: 0.015	
			X3:Ca	B3:-0.415	
			X4:CRP	B4: 0.006	
			X5:Ferritin	B5: 0.001	
Mortality based on correlation coefficients	Z = B0+B1X1+B2X2+B3X3+B4X4 (M2 Mortality)	(B0): (-1.342)	X1:D-dimer	B1:0.064	Accuracy 77+/-5%; sensitivity 68.6%; specificity 69.3%; PPV 57.2%; NPV 78.8%
			X2:Urea	B2: 0.017	
			X3:CRP	B3: 0.006	
			X4:IL6	B4: 0.008	

## Discussion

In the fight against COVID-19, knowledge of biochemical parameters and their correlation with disease severity is crucial to early intervention. A scientific model to triage patients by severity will reduce morbidity and mortality. We compared biochemical parameters in Indian COVID-19 patients during the first and second waves of the pandemic, between those admitted to the ICU and non-ICU, and among discharged and deceased patients. These biochemical parameters were noted at the time of admission and also before discharge or death. This was done to identify the best set of biochemical parameters, which were then used to construct the predictive model.

Patients admitted in phase 2 had higher NL ratios and lower CRPs than in phase 1 during admission. Patients who got discharged in phase 2 had a higher NL ratio and ALT than those in phase 1. In developing countries like India, the overuse of corticosteroids may be due to the benefits observed during phase 1. Corticosteroids cause lymphocytopenia and thus increase the NL ratio [12]. The CRP is a marker of inflammation and was higher in phase 1 than phase 2, possibly due to COVID-19 patients' pre-hospital use of steroids [13]. Among the patients who succumbed to COVID-19, phase 1 showed higher creatinine and urea values before death than in phase 2. The COVID-19 infection affects the kidneys and lungs. Acute kidney injury due to COVID-19 increases urea and creatinine [14]. In phase 2, the use of corticosteroids possibly reduced COVID-19-related inflammation and kidney injury, thus lowering urea and creatinine when compared to phase 1. Among the deceased patients, phase 2 had higher ferritin and procalcitonin levels than phase 1. These corroborate the reality that the COVID-19 second wave was more severe [15,16].

Next, we analyzed the difference in biochemical parameters between ICU and non-ICU admissions. Ten parameters out of 14 differed significantly between ICU and non-ICU admissions. The ICU patients had severe COVID-19 infections, so their biochemical parameters were higher than those of non-ICU patients except for Ca and albumin, which correlated negatively with the severity of the infection. The levels went down in both groups at the time of discharge but were still higher in ICU patients, especially ferritin, due to the very high levels present at admission. Further, we wanted to see the differences in the biochemical parameters among the patients who survived COVID-19 and those who succumbed to it. Eleven biochemical parameters out of 14, measured at admission, were significantly different between COVID-19 survivors and the deceased. The NL-ratio, D-dimer, urea, LDH, IL-6, CRP, and ferritin were significantly higher in patients who succumbed. Among non-ICU patients upon admission, we observed significant differences in eight out of 14 biochemical parameters between those who survived COVID-19 and those who succumbed to it, indicating severe illness in the affected patients. The differences expanded to encompass 12 out of 14 parameters when comparing the values of these parameters before discharge or death, highlighting a notable shift in health status.

Among the ICU patients at the time of admission, we found significant differences in three of the 14 biochemical parameters between those who survived COVID-19 and those who expired, namely Na, urea, and LDH. Urea and LDH were significantly higher in those who succumbed, but Na was within the normal range in both groups, though the difference was significant. Studies have shown that COVID-19 patients with higher LDH levels are at increased risk of death [17,18]. The notable variances in biochemical parameters extended to 10 of the 14 parameters analyzed when comparing the values obtained before discharge or death.

Among all these biochemical parameters compared between ICU/non-ICU and discharge and death, nine biochemical parameters were common and had significantly higher values among the deceased patients when compared with the survivors, taken just before death or discharge. This indicates that the WHO classification [4] of the patients according to severity is robust in categorizing patients for ICU but doesn’t hold good for the non-ICU category or predicting mortality. Therefore, patient categorization should also consider these eight biochemical parameters. Significant differences in the 12 biochemical parameters were measured just before discharge or death, which further reinforces our observation. During their non-ICU stay, the condition of some patients worsened. The deterioration may have been mitigated by ICU admissions. Those deceased non-ICU patients who were admitted according to the clinical admission criteria should have been admitted to the ICU according to our model. In a pandemic, once a patient's condition worsens, they should be shifted to the ICU. However, this is subject to the availability of beds in the ICU. Our model predicted ICU admission at the time of admission for these deceased non-ICU patients.

In a study by Kumari et al., high levels of laboratory parameters such as IL-6, LDH, prothrombin time (PT), INR, activated partial thromboplastin time (aPTT), ferritin, WBC count, and D-dimer were significantly associated with poor outcomes [19]. Similar findings were seen in our study. Age, high-sensitivity CRP level, lymphocyte count, and D-dimer level among COVID-19 patients at admission were found to be informative of the outcomes and aided our model building [20]. In another study, it was found that the variables that exerted the greatest influence on mortality prediction were ferritin, fibrinogen, D-dimer, platelet count, CRP, PT, invasive mechanical ventilation (IMV), PaFi (PaO2/FiO2), LDH, lymphocyte levels, aPTT, BMI, creatinine, and age [21].

The analysis of our data in this study helped formulate the prediction models. To further establish our observation, we tried to predict the cut-off values of these biochemical parameters to predict ICU admission and also developed a predictive model for triage. We wanted to find the cut-off values of biochemical parameters at the time of admission to be able to predict COVID-19 mortality. In our data set, ICU admission and mortality were highly correlated, so these cut-offs can be used for ICU admission. Table 5 and Figure 1 show biochemical variable ROC curves. The NL ratio, D-dimer, urea, LDH, IL-6, and procalcitonin were highly significant. We wanted to know, for patients admitted to the ICU, if the biochemical parameters at the time of admission and mortality are correlated. Table 1 shows the correlation coefficients of combined biochemical parameters measured at admission in phases 1 and 2. The NL ratio, D-dimer, urea, LDH, IL-6, CRP, ferritin, and procalcitonin correlated positively and significantly with ICU admission. The ICU admission was linked to higher parameters. Albumin and Ca were negatively and significantly correlated with ICU admission. The NL ratio, urea, D-dimer, creatinine, LDH, IL-6, CRP, ferritin, and procalcitonin were positively and significantly correlated with disease mortality. Higher values of these parameters were associated with COVID-19 mortality. Disease mortality due to COVID-19 showed a significant inverse correlation with albumin and Ca levels in our study. There were many parameters with statistically significant differences, and though they were in the normal range, their clinical significance is debatable. We statistically analyzed various biochemical parameters between various groups to ensure the best possible parameters were chosen for model prediction. 

We developed four biochemical prediction models, two for ICU admission and two for mortality, to triage patients by severity, and we can use these probabilities to decide whether a patient should go to the ICU or not based on bed availability. Twenty-one clinically admitted (per WHO categorization) non-ICU patients succumbed to COVID-19 in our dataset. On applying our ICU prediction model, 11 patients had a high probability of ICU admission. Among the remaining 10, seven had a high probability of mortality. Had we used both models simultaneously, we could have sent a total of 18 of those 21 patients to the ICU for better management.

We applied our ICU prediction model to our study population of 324 patients. Our model predicted 117 (36%) ICU admissions and 207 (64%) non-ICU admissions. Of these 117 patients, 82 (70%) succumbed to COVID-19. Of the 82 that expired, 65 (80%) patients had a high mortality risk, while 17 had a low mortality risk. However, these 17 patients already had a high ICU admission probability. This implies that both models need to be used simultaneously for better prediction. Among those 207 non-ICU admissions, 138 (67%) patients had a low mortality probability, and 69 (33%) patients had a high mortality probability. Twenty-one (30%) of those 69 patients died, and 18 (85%) of the deceased 21 were admitted to the non-ICU. Of the 138 patients with low mortality, 18 (13%) died, 15 of the deceased 18 patients were admitted to the ICU, and the remaining three were in the non-ICU.

Twenty-six patients were discharged from the ICU. Our model predicted 17 ICU admissions for these 26 discharged patients from among 126 ICU admissions. Seven of the remaining nine patients had low mortality probabilities. Effectively, seven of these 26 patients had a low probability of both ICU admission and mortality. Hence, these seven patients could have been admitted to the non-ICU, and in a resource-poor setting, those seven ICU beds could have been utilized in a much better way.

So to conclude, our model prediction capability is superior in the prediction of non-ICU admission compared to the ICU admission of COVID-19 patients. The algorithm that we suggest in this paper is: (a) patients coming to the emergency (n = 324; 'n' denotes the number of patients in that category after applying our model) are (b) evaluated based on the WHO criteria, i.e., mild (PaO2/FIO2 = 201-300 mmHg), moderate (PaO2/FIO2 = 101-200 mmHg with positive end-expiratory pressure (PEEP) ≥5 cm of H2O), and severe (PaO2/FIO2 ≤100 mmHg with PEEP ≥5 cm of H2O), with (bI) severe cases receiving ICU admission (n = 126), (bII) mild/moderate being suggested to the non-ICU admission (n = 198). Now, we apply our ICU model equation to (bII), and we get admission probability (c) < 50 (n = 158) and (d) >50 (n = 40). Those with a probability of more than 50 will be admitted to the ICU (d). Next, we apply our mortality model to (c). Those with a prediction of mortality probability > 50 (n = 43) will be admitted to the ICU, and those with < 50 (n = 115) will be non-ICU admissions (three deaths). 

Patients can be initially classified as per WHO criteria into mild, moderate, and severe based on the clinical signs and symptoms at the presentation. The severe patients are directly admitted to the ICU. When the clinical criteria suggest non-ICU admission, the ICU admission model should be applied, and if the probability is greater than 50%, the patient should be sent to the ICU. If the ICU admission probability is less than 50%, then the mortality model should be applied, and if the mortality probability is more than 50%, then the patient should be sent to the ICU. But when in both models the probability is less than 50%, then the patient should be managed in the non-ICU. This suggests that the predictive power of our model is superior for non-ICU admissions as compared to ICU admissions. This is especially useful in resource-poor countries such as India and enables better utilization of ICU beds.

Our model analyzes parameters mostly available in tertiary healthcare settings and provides straightforward formulas for complex variables so they can be calculated from simple variables in primary healthcare centers (PHCs). Thus, if a patient goes to a PHC and both ICU and mortality probability are over 50%, they will be transferred to a nearby tertiary healthcare center without delay, thus potentially saving lives.

Limitations

The development of this model is based on the collection of biochemical parameters from a single tertiary health center. A larger sample size would have been better to form a predictive model with even higher accuracy. Also, we have not looked into the individual comorbidities of the patients.

## Conclusions

The COVID-19 pandemic caused a severe and devastating effect around the world, and assessing its severity and management has been a dilemma for physicians. Biochemical parameters can be used to predict COVID-19 disease severity. We have constructed a formula to predict IL-6 and procalcitonin values from simple biochemical parameters. We have also tried to develop an ICU admission and mortality prediction model using these biochemical parameters. By using our model, we have correctly placed 92 out of 96 patients in the study into the correct management category. Furthermore, had our model been applied, it is assumed that we could have potentially saved 17 of the 21 patients we lost. This approach will pave the way for the development of similar models for other diseases and promote the use of machine learning to prognosticate patient outcomes. This study is probably the first of its kind to predict the triage of infective patients based on biochemical parameters.
